# Stable Isotope Biogeochemistry of Seabird Guano Fertilization: Results from Growth Chamber Studies with Maize (*Zea Mays*)

**DOI:** 10.1371/journal.pone.0033741

**Published:** 2012-03-30

**Authors:** Paul Szpak, Fred J. Longstaffe, Jean-François Millaire, Christine D. White

**Affiliations:** 1 Department of Anthropology, The University of Western Ontario, London, Ontario, Canada; 2 Department of Earth Sciences, The University of Western Ontario, London, Ontario, Canada; New York State Museum, United States of America

## Abstract

**Background:**

Stable isotope analysis is being utilized with increasing regularity to examine a wide range of issues (diet, habitat use, migration) in ecology, geology, archaeology, and related disciplines. A crucial component to these studies is a thorough understanding of the range and causes of baseline isotopic variation, which is relatively poorly understood for nitrogen (δ^15^N). Animal excrement is known to impact plant δ^15^N values, but the effects of seabird guano have not been systematically studied from an agricultural or horticultural standpoint.

**Methodology/Principal Findings:**

This paper presents isotopic (δ^13^C and δ^15^N) and vital data for maize (*Zea mays*) fertilized with Peruvian seabird guano under controlled conditions. The level of ^15^N enrichment in fertilized plants is very large, with δ^15^N values ranging between 25.5 and 44.7‰ depending on the tissue and amount of fertilizer applied; comparatively, control plant δ^15^N values ranged between −0.3 and 5.7‰. Intraplant and temporal variability in δ^15^N values were large, particularly for the guano-fertilized plants, which can be attributed to changes in the availability of guano-derived N over time, and the reliance of stored vs. absorbed N. Plant δ^13^C values were not significantly impacted by guano fertilization. High concentrations of seabird guano inhibited maize germination and maize growth. Moreover, high levels of seabird guano greatly impacted the N metabolism of the plants, resulting in significantly higher tissue N content, particularly in the stalk.

**Conclusions/Significance:**

The results presented in this study demonstrate the very large impact of seabird guano on maize δ^15^N values. The use of seabird guano as a fertilizer can thus be traced using stable isotope analysis in food chemistry applications (certification of organic inputs). Furthermore, the fertilization of maize with seabird guano creates an isotopic signature very similar to a high-trophic level marine resource, which must be considered when interpreting isotopic data from archaeological material.

## Introduction

Seabird excrement (guano) was arguably the most economically significant organic fertilizer in the world prior to the twentieth century. The guano was mined from small, nearshore islands off the arid western coast of South America in the Peru-Humboldt upwelling region. The guano islands of Peru and Chile are typically composed of rocky cliffs essentially devoid of vascular plants, with a relatively small number of fauna (ants, spiders, scorpions, lizards) that are supported by allocthonous inputs from the guano birds (guano, carcasses, feathers, eggshells) [1]. Because the region receives virtually no precipitation, the guano accumulates in sedimentary layers. The once thick deposits of seabird guano (>50 m in some cases) were mined extensively during the guano boom of the 1800 s, and today the islands rarely have more than several years worth of droppings accumulated [1]. The trade in guano peaked during the middle of the nineteenth century, with 20 million tons being exported to Europe and North America between 1848 and 1875 [2]. The popularity of guano with European and North American farmers waned in the latter part of the nineteenth century for a number of reasons, including: increasing guano prices, irregular availability, unsuitability for particular crops (especially turnips), a dwindling supply, and the development of the chemical fertilizer industry [3]. In recent years, however, there has been a resurgence in its popularity (particularly in horticulture) as worldwide demand for organically grown produce has increased [4], [5]. The importance of guano as a fertilizer prior to the nineteenth century is less well known, but is mentioned by Spanish chroniclers and in colonial administrative documents [6], [7]. On this basis, some have suggested that it may have been of some importance in prehispanic agriculture [8], [9].

From an ecological perspective, the importance of ornithogenic nitrogen to marine and terrestrial ecosystems has long been recognized [10]–[12]. A number of studies conducted in tropical, temperate, subpolar, and polar regions have shown that seabird guano alters the concentration of soil nutrients (particularly NH_4_^+^, NO_3_^−^, PO_4_^3−^, K^+^, Mg^2+^), plant tissue nutrients (N, P, K), and plant productivity [13]–[21]. Seabird guano may also affect the diversity of plant species present, though results from such studies are inconsistent [21]. Numerous factors other than the presence of guano may also affect the chemistry, physiology, and ecology of plants growing within or near seabird colonies. In field studies it is often difficult, or impossible, to rule out the effects of these factors, which include: physical disturbance caused by birds such as plant clipping or trampling [22], [23], deposition of seabird carcasses, feathers and eggshells [24]–[26], and avian-aided seed dispersal [27].

Particularly large ^15^N enrichments in soils, plants, and animals (5–40‰) have been recorded in and around seabird nesting sites, allowing for the relative contribution of avian-derived nutrients to be assessed (Table 1). Despite this large body of literature, there have been no investigations that examine the biogeochemical effects of seabird guano on the western coast South America, with the majority of studies focusing on Oceania, Japan, California, and Antarctica [21]. Furthermore, no studies have addressed the isotopic biogeochemistry of seabird guano from an agricultural or horticultural standpoint. The purpose of this study, therefore, is to assess the isotopic and vital effects of Peruvian seabird guano fertilization on maize (*Zea mays*) under controlled conditions. In particular we examine the extent of the enrichment in plant ^15^N resulting from guano fertilization.

**Table 1:** Summary of studies examining the effects of seabird guano on the isotopic composition (δ^15^N) of plants and soils.

Location	Bird Species	Guano δ^15^N (‰, AIR)	Plant δ^15^N (‰, AIR)		Plant δ^15^N (‰, AIR)		Reference
			Bird	Non-bird	Bird	Non-bird	
California	Mixed	–	36.4±2.7	5.3±0.9	33.6±2.0	7.2±1.2	[13]
California	Mixed	–	24.3 to 24.5	6.8 to 7.8	–	–	[173]
Falkland Islands and Antarctica	Mixed	10.9±1.9	−1.8 to 15.8	−7.9 to 7.6	14.0±0.3	0.0 to 9.7	[174]
Antarctica	Snow petrel	–	−3.1 to 25.8	−17.6 to −0.5	13.1 to 25.9	−13.4 to −1.0	[175]
Australia	Gentoo penguin	14.6	7.2 to 18.8	−9.8 to −0.7	–	–	[176]
North Africa	Various gulls	–	9.8 to 17.4	–	10.5 to 13.4	16.8 to 20.8	[51]
New Zealand	Westland petrel	–	–	–	14.1±0.3	–	[177]
New Zealand	Mixed	–	−3.9 to 9.1	–	–	–	[178]
New Zealand	Sooty shearwater	7.7	14.2±3.1	−6.1±1.7	–	–	[179]
Japan	Great cormorant	–	16.4 to 16.9	−2.5±0.6	10.6 to 16.0	0.4±0.3	[180]
Japan	Great cormorant	13.2±1.3	10.0 to 14.7	−2.3 to 6.8	–	–	[181]
Sweden	Great cormorant	–	13.6±1.7	1.7	–	–	[182]
New Zealand	Mixed	–	4.6 to 6.7	14.4 to 15.9	10.2±1.0	16.2±0.3	[183]
Fiji	Mixed	39.1 to 50.1^a^	13.6 to 36.7	−1.3 to 0.8	15.1 to 31.6^a^	−4.1 to −1.3^b^	[184]
Fiji	Mixed	14.9 to 23.3^b^	–	–	13.5 to 33.0^b^	–	[184]
Japan	Black-tailed gull	10.2 to 10.5	3.9 to 14.6	−4.1 to −2.1	10.1 to 43.3^a^	−4.3 to −2.9^b^	[185]
Japan	Black-tailed gull	–	–	–	−0.2 to 33.7^b^	–	[185]
Japan	Black-tailed gull	9.1 to 12.8	–	–	18.5 to 44.1^a^	–	[53]
Japan	Black-tailed gull	–	–	–	−4.1 to 42.2^b^	–	[53]
Japan and Antarctica	Penguin and gull	8.0 to 9.4	13.6 to 38.1	–	–	–	[186]
Antarctica	Penguin	7.4	–	–	32.1	–	[75]
Japan	Mixed	–	–	–	9.1 to 37.9	−4.6 to 8.6	[54]
New Zealand	Rockhopper penguin	7.0±0.4	–	–	23.8±3.3	−0.5±0.2	[187]
Australia	Mixed	9.9	9.5±2.2	7.0±2.6	–	–	[55]
California	Mixed	–	27.2 to 27.3	8.3 to 9.5	28.3±5.4	–	[188]
Pribilof Islands	Mixed	12.5	22.0	11.3	–	–	[162]
California	Mixed	–	–	–	35.6±2.6	7.5±0.3	[56]
Pacific (Palmyra Atoll)	Mixed	13.9	14.0±1.4	9.3±0.9	16.2±0.3	11.0±0.7	[189]
Antarctica	Penguin	20.9±4.2	–	–	10.4±3.1	–	[190]

a NH_4_^+^.

b NO_3_^−^.

Plants are capable of utilizing several different soil N sources, both organic (amino acids) and inorganic (NH_4_^+^, NO_3_^−^, N_2_). From a biogeochemical perspective, the uptake, assimilation, and allocation/reallocation of N compounds are all significant. Uptake of NO_3_^−^ in plant root cells occurs through at least three different NO_3_^−^ transport systems [28]. Once inside the root, NO_3_^−^ can be assimilated into organic N, or translocated to the shoot for assimilation by nitrate reductase (NR), nitrite reductase (NiR), and glutamine synthetase (GS) [29]. Little or no fractionation of ^15^N is reported to be associated with the uptake of NO_3_^−^ [30]–[32]; fractionation of ^15^N does not appear to vary with respect to source [NO_3_^−^] [33]–[35]. Some variability in fractionation is associated with NR activity, and it has been difficult in some cases to differentiate between isotopic fractionation associated with N uptake and assimilation, respectively [36]. Ledgard et al. [37] report the fractionation for the entire process to be −15‰, while a range of 0 to −19‰ is reported by Robinson [38].

NH_4_^+^ is taken up by plants via high or low affinity transporters depending on extracellular [NH_4_^+^] [39]. NH_4_^+^ is assimilated into organic N only in the roots via GS and most estimated Δ^15^N values for NH_4_^+^ uptake and assimilation fall between −5 and −20‰ [38], [40]. Unlike NO_3_^−^, however, there are substantial differences in Δ^15^N with source [NH_4_^+^]. For example, in two different rice cultivars, Yoneyama et al. [33] found Δ^15^N for NH_4_^+^ uptake to be −6.1 to −12‰ at low source [NH_4_^+^], and −13.4 to −28.9‰ at high source [NH_4_^+^].

## Materials and Methods

### Materials

All plants were grown in a walk-in growth chamber at the Biotron Centre for Experimental Climate Change Research at the University of Western Ontario. The substrate utilized for all treatments was Pro-mix® for containers (75–85% sphagnum moss, 15–25% perlite and limestone). Peruvian seabird guano (Guano Company International, Cleveland, Ohio, United States) was obtained from an organic gardening outlet. The nitrogen content of the guano was reported to be 10% and determined to be 11.2±0.2% based on five analyses of dried, powdered guano as described for plant samples below. The ‘Early Sunglow’ maize cultivar was used (*Zea mays* cv. Early Sunglow, Lot E1, 2010, Ferry Morse, Fulton, Kentucky, United States) for all experiments because it is a relatively small variety of maize that accommodated physical restrictions on plant height imposed by the growth chamber.

### Growth Chamber Conditions

Growth chamber temperature was 25/18°C (day/night), with a photoperiod of 13 h provided by 185 W fluorescent bulbs. Relative humidity was set at 80% for the first four daylight hours, and 60% for the remainder of the day. These conditions were monitored electronically, and did not deviate from these parameters for the duration of the experiment.

### Maize Germination Experiment

Guano (well-mixed with soil) was applied to 1.2 L plastic containers (1.0 L of soil) in the following amounts: 0 g, 1.0 g, 2.5 g, 5.0 g, 7.5 g, 10.0 g and 15.0 g. Six replicates of each treatment were prepared. One hour after addition of the guano, maize seeds were planted ∼2.5 cm below the surface in the containers. Emergence and growth of the plants were recorded every 2–3 days for 35 days.

### Maize Fertilization Experiment

Fifteen maize seeds were planted ∼2.5 cm below the surface in 1.2 L plastic containers (1.0 L of soil). At this time, guano was mixed with soil in free-draining (perforated at the base) 18.9 L plastic buckets containing 16 L of soil in the following amounts: 0 (C0), 80 g (G1, 5 g guano/L), 160 g (G2, 10 g guano/L). Five replicates of each treatment were prepared. Maize is typically fertilized prior to planting, and sometimes again approximately three weeks after emergence, although this second application is uncommon [41]. To avoid complications associated with additional fertilizer applications, only one fertilizer application was employed. After germination (7 days after sowing) maize plants were moved into the 18.9 L plastic buckets. Plants were watered every 2–3 days and the height and general growth of the plants was monitored. Distal leaf samples (∼3 cm×6 cm) were taken at 30 and 75 days after planting (d). Plants at 30 d were characterized by only vegetative growth, while plants sampled at 75 d had begun reproductive growth (tassels fully emerged, silks beginning to appear). Anthers were sampled at 75 d. At completion of the experiment (115 d), the following tissues were sampled: leaves, grains, roots, and stalks. All buckets were relocated randomly within the growth chamber five times (30, 45, 60, 75, 100 d) during the course of the experiments to account for any micro-variations in light, temperature or humidity, although such changes were not expected.

### Stable Isotope Analysis

All plant materials were stored at −25°C following sampling until needed for analysis. Samples were then dried at 90°C under normal atmosphere for 72 hours, ground using a Wig-L-Bug (Crescent, Lyons, Illinois, United States) and the resulting powders stored at room temperature in sealed glass vials. Isotopic compositions (δ^13^C and δ^15^N values determined separately) and relative percentages of carbon and nitrogen were determined using a Delta V isotope ratio mass spectrometer (Thermo Scientific, Bremen, Germany) coupled to an elemental analyzer (Costech Analytical Technologies, Valencia, California, United States). For the analysis of δ^15^N, excess CO_2_ was removed using a Carbo-Sorb trap (Elemental Microanalysis, Okehampton, Devon, United Kingdom). Sample reproducibility was ±0.09‰ for δ^13^C and ±0.90% for %C (6 replicates), and ±0.12‰ for δ^15^N and ±0.10% for %N (24 replicates). A δ^15^N value of 20.31±0.18‰ was obtained for 37 analyses of IAEA-N2, which compared well with its accepted value of 20.30‰. A δ^13^C value of −29.87±0.29‰ was obtained for 11 analyses of NBS-22, which compared well with its accepted value of −30.00‰.

### Statistical Analyses

Comparisons between treatments and between organs were completed using one-way analysis of variance (ANOVA). Levene's test was used to assess homogeneity of variance; if variance was homoscedastic, a *post hoc* Tukey's honestly significant difference (HSD) test was applied and if variance was not homoscedastic, a *post hoc* Dunnett's T3 test was applied. All statistical analyses were conducted at a significance level of 5% (*p*<0.05). All statistical analyses were performed in SPSS 16 for Windows.

## Results and Discussion

### Maize Germination and Seedling Establishment

**Figure 1:**
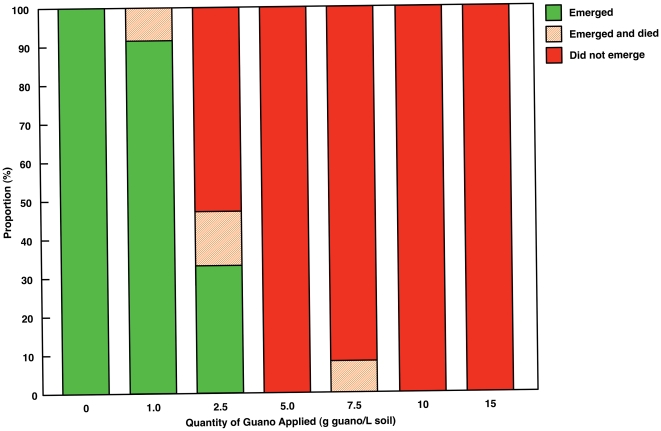
Relative percentages of seedlings that germinated and emerged with differing amounts of seabird guano applied.

All unfertilized plants germinated and commenced normal growth (Figure 1). There was a clear trend towards the inhibition of germination and seedling emergence with increasing rate of guano applied (Figure 1). It is apparent that the presence of seabird guano in the soil has the potential to inhibit germination and that this effect is concentration dependent. Ishida [42] found lower germination rates in oak and pine trees within, compared to outside of, cormorant colonies but did not offer a detailed explanation for this pattern. Mulder and Keall [43] also found that seabird guano negatively affected seed germination and seedling survival. Germination inhibition with increasing concentrations of guano probably results from a number of factors, including reduced soil pH and the presence of a high concentration of soluble salts, both of which are characteristic of ornithogenic soils [20]. Very high concentrations of NO_3_^−^ and especially NH_4_^+^ are also characteristic of ornithogenic soils and these characteristics can inhibit maize germination [44], with the early stages of growth being the most detrimental for plants under NH_4_^+^ stress [45], [46].

### Vital Effects of Guano Fertilization

**Figure 2:**
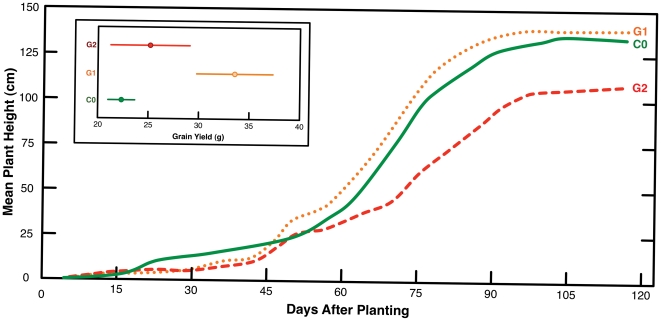
Maximum heights of maize plants throughout experiment. Harvest occurred at 115 d. Inset: grain yield for each experiment.

Plant growth was strongly inhibited in the heavy guano treatment (G2). Maximum plant heights were significantly lower in G2 compared to C0 (*p* = 0.02) and G1 (*p* = 0.008) (Figure 2). While the G1 plants did not attain greater maximum heights than the C0 plants (*p* = 0.83), they yielded significantly more grain (*p* = 0.004). The G2 plants yielded less grain than the G1 plants (*p* = 0.03) and more grain than the C0 plants, although this difference was not statistically significant (*p* = 0.42) (Figure 2).

In this study, we observed a positive influence of guano on maize growth at moderate concentrations (G1), but a negative influence at high concentrations (G2). A number of studies have found that plant abundance and/or species richness tends to be lower within seabird colonies, but is often higher in areas in relatively close proximity to the colonies [14], [22], [47], [48].

Very high levels of soil P can have deleterious effects on plant growth [49]. Ornithogenic soils may contain fifty times more available phosphorous than normal, but the P salts in bird excrement tend to be immobile in soil because of their limited solubility, making them generally unavailable for uptake by plants [4], [50], [51]. It is thus unlikely that the reduced growth observed in the G2 plants is the result of P toxicity. The most likely cause for the reduced growth of the G2 plants is NH_4_^+^ toxicity.

Very high [NH_4_^+^] is a ubiquitous trait of ornithogenic soils [52]–[56]. High soil NH_4_^+^ can negatively impact plants in several ways: (1) soil acidification, particularly of the rhizosphere [57], ‘scorching’ of root hairs [46]; (2) accumulation of free NH_4_^+^ in plant tissues, which has the capacity to uncouple plastid energy gradients [46]; (3) assimilation of NH_4_^+^ in the roots and associated translocation of carbon skeletons from the shoot, which is metabolically expensive and places ‘carbon stress’ on roots [58]; (4) suppression of the expression of certain proteins (aquaporins), which can have detrimental effects on the uptake of water [59]; and (5) the influx and efflux of NH_4_^+^ through root cells, which is associated with a very high metabolic cost when source [NH_4_^+^] is high [60].

Both the G1 and G2 plants exhibited significantly reduced growth compared to the control plants for the first 45 days of the experiment (Figure 2; *p* = 0.01), but this trend did not continue as the G1 plants produced the greatest yields, and had similar maximum heights to the control plants. This is likely the result of initially very high soil [NH_4_^+^], which negatively impacted the growth of the fertilized plants, followed by increased soil NO_3_^−^ resulting from nitrification of guano-derived NH_4_^+^. When plants largely supplied with NH_4_^+^ as an N source are supplemented with NO_3_^−^, NH_4_^+^ uptake is suppressed and plants are able to resume normal growth [58]. The fact that the G2 plants still produced grain even though they were characterized by reduced heights and less above–ground biomass than either the control or G1 plants suggests that there was some acclimatization of these plants to the high [NH_4_^+^], and/or nitrification was substantially delayed and [NH_4_^+^] remained high in the soil for a much longer period of time. Schortemeyer et al. [46] observed a similar result in maize plants grown with NH_4_^+^ as the sole N source.

The effects of guano on plants are difficult to generalize. There is considerable variability at the community level and also within a community in accordance with plant physiology (nutrient demands, salt tolerance) at the species level [47], [52]. Even within maize there are differences in NH_4_^+^ tolerance, with some varieties being able to survive higher concentrations than others [46]. Therefore, it cannot be assumed that the results of this study are directly applicable to all maize varieties.

### Nitrogen Isotope Composition of Seabird Guano

Most inorganic N fertilizers have δ^15^N values close to 0‰, with organic fertilizers generally having highly variable but positive δ^15^N values (Table 2). The δ^15^N value of the seabird guano used in this experiment was 26.7±0.6‰ (5 analyses), which is much higher than any other organic fertilizer analyzed to date. This is the product of avian nitrogen metabolism and excretion, which is quite different than in mammals, combined with the high trophic position of the guano-producing birds. Guano contains 9–21% nitrogen, which is composed primarily of uric acid (∼80%), with smaller amounts of protein (∼10%), ammonia (∼7%), and nitrate (∼0.5%) [23], [61]–[67]. In addition, guano contains ∼4% phosphorous (∼50% of which is PO_4_^3−^) and 2% potassium [62], [67], [68].

**Table 2:** δ^15^N values of organic and inorganic fertilizers.

Type	Fertilizer	Fertilizer δ^15^N (‰, AIR)	Reference
Organic	Blood	6.0±1.3	[191]
	Bonemeal	4.9±0.3	[191]
	Cattle manure	5.0±0.8	[192]
	Cattle manure	2.9±0.5	[193]
	Cattle manure	4.5	[133]
	Cattle manure	3.1±0.2	[85]
	Chicken manure	6.2±1.9	[191]
	Fishmeal	7.1±3.6	[191]
	Hoof and horn	6.4±0.2	[191]
	Livestock manure	8.8±4.4	[191]
	Livestock manure	8.7±0.2	[132]
	Pig manure	13.9	[102]
	Pig manure	16.9	[194]
	Pig manure	11.3	[133]
	Pig manure	6.5	[133]
	Pig manure	16.4	[195]
	Poultry manure	8.6±0.3	[132]
	Poultry manure	2.7	[133]
	Seabird guano	26.7±0.6	This study
	Seaweed	2.5±1.5	[191]
	Various composts	17.4±1.2	[196]
Inorganic	(NH_4_)_2_H_2_PO_4_	−0.6±0.4	[191]
	(NH_4_)_2_SO_4_	1.7±3.4	[191]
	(NH_4_)_2_SO_4_	−1.6	[133]
	(NH_4_)_2_SO_4_	−2.6	[197]
	KNO_3_	−1.2±0.3	[191]
	NH_4_NO_3_	−1.3	[128]
	NH_4_NO_3_	−0.6±1.7	[191]
	NH_4_NO_3_	−1.7	[133]
	Urea	−2.4±2.1	[191]
	Urea	−1.7	[133]
	Urea	−0.7	[195]

**Figure 3:**
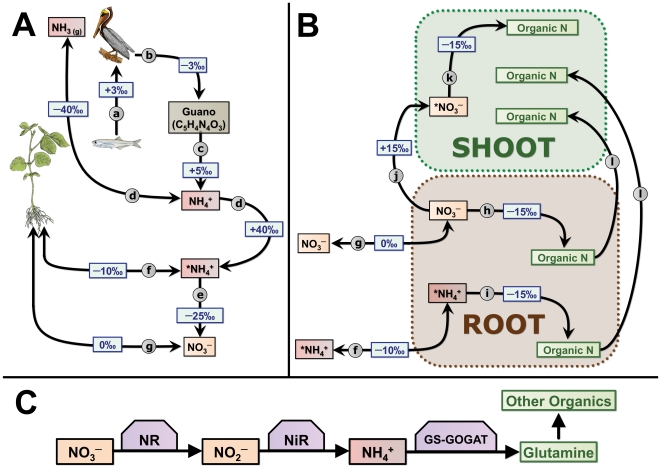
Simplified schematic of fractionation factors associated with decomposition and uptake of seabird guano N. **A**) Simplified pathway for guano-derived nitrogen. (**a**) Incorporation of dietary N into consumer tissue N. Tissue–diet fractionation for birds has been calculated to be ∼3‰ for most tissues [160], [161]. (**b**) Excretion of dietary N as uric acid. Wainright et al. [162] found bulk guano to be depleted of ^15^N by 2.5‰ relative to seabird blood. Moreover, Mizutani et al. [75], [163] and Bird et al. [164] found δ^15^N of uric acid to be very similar to bulk guano δ^15^N. (**c**) Conversion of uric acid to NH_4_^+^, according to the experiment performed by Mizutani et al. [163]. (**d**) Ammonia volatilization. Many studies have found this process to be associated with a large equilibrium fractionation that concentrates ^15^N in the remaining substrate (*NH_4_^+^ in the diagram) [54], [75], [165]. (**e**) Nitrification. The fractionation factor for the entire process of nitrification in the soil (NH_4_^+^→NO_2_^−^→NO_3_^−^) is estimated to be between −12 and −35‰ [38], [166], [167]. (**f**) Uptake of NH_4_^+^ is associated with a nitrogen isotope fractionation ranging from −6 to −30‰ and appears to depend on the concentration of the source NH_4_^+^ [33], [168]. (**g**) Uptake of NO_3_^−^ by the plant does not appear to be associated with any fractionation [33], [169], [170]. Both NO_3_^−^ and NH_4_^+^ may be effluxed from the plant, passively and in some cases actively [171]. **B**) (**h**) NO_3_^−^ assimilation into organic N occurs in the root by the NR-NiR (nitrate reductase-nitrite reductase) and GS-GOGAT (glutamine synthetase–glutamine:oxoglutarate aminotransferase) pathways (see Figure 4C). The reduction of NO_3_^−^ to NH_4_^+^ is associated with a fractionation factor of −15‰ [37], [172]. (**i**) NH_4_^+^ assimilation occurs in the root via the GS-GOGAT pathway and is associated with a fractionation factor of −10 to −15‰ [40], [94]. (**j, k**) NO_3_^−^ may also be mobilized to the shoot for assimilation. In this case, this NO_3_^−^ pool has already been exposed to NO_3_^−^ assimilation in the root and is enriched in ^15^N [95]. Therefore, organic N formed from NO_3_^−^ in the shoot (*NO_3_^−^) will have a higher δ^15^N value than organic N formed from NO_3_^−^ in the root. (**l**) Organics may be moved between the root and shoot. **C**) Simplified schematic for the assimilation of N by plants. For a more detailed description see Miller and Cramer [171]. All fractionation factors are approximate values representing medians of ranges, which may be large (see text for discussion).

A simplified pathway for guano nitrogen, with associated nitrogen-isotope fractionation factors, is shown in Figure 3. The principal producers of guano on the western coast of South America are the Peruvian booby (*Sula variegata*), brown pelican (*Pelecanus occidentalis thagus*), and guanay cormorant (*Phalacrocorax bougainvilli*) [1], [12]. These birds, and similar species, feed at high trophic levels, and typically have tissue δ^15^N values in the range of 17 to 20‰ [69]–[71], suggesting a δ^15^N_diet_ of 14 to 18‰ assuming a diet–tissue fractionation of 3–4‰ for δ^15^N [72]. Thus, the high trophic level of the birds only partially explains the very high δ^15^N_bulk guano_ of 26.7‰.

After deposition in the soil, the uric acid in guano is rapidly mineralized to NH_4_^+^, and this process occurs much more rapidly in the presence of water [68], [73], [74]. Based on results presented by Mizutani and Wada [65], uric acid quickly decomposed (75% in ten days) in soil, but the δ^15^N value of the remaining uric acid was unchanged. A very large isotopic fractionation (−40 to −60‰) occurs during NH_3_ volatilization, leaving the remaining soil NH_4_^+^ highly enriched in ^15^N [38], [75]. Ammonia volatilization is largely responsible for the high δ^15^N values in ornithogenic soils and in some cases, seabird guano (Table 1). The relatively high δ^15^N value of the guano utilized in this study suggests that some of the NH_4_^+^ in the guano had been subject to volatilization prior to deposition in the soil during the experiment; similar observations have been made concerning other avian manures [76].

### ^15^N Enrichment in Guano Fertilized Plants

**Figure 4:**
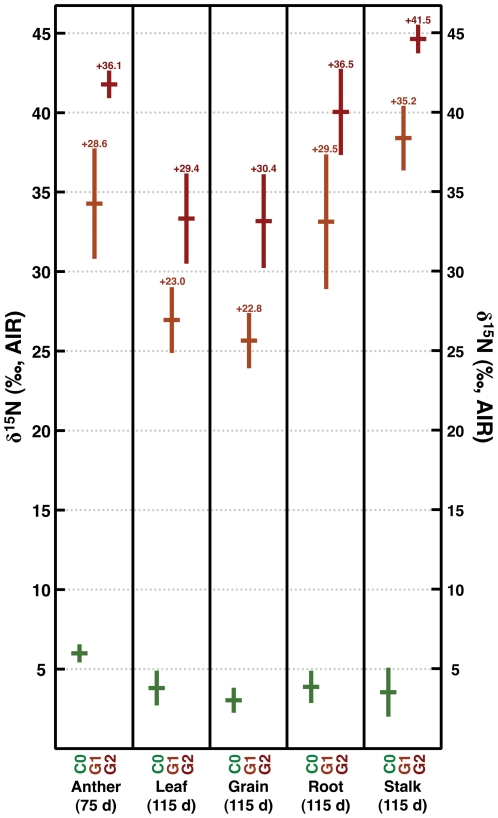
Mean nitrogen isotope composition of maize organs; horizontal bars represent means, vertical bars represent standard deviations. Values above G1 and G2 maize represent differences in nitrogen isotopic composition relative to C0 maize.

Plant isotopic compositions are summarized in Table 3; raw data are presented in Table S1. Plant organs of fertilized plants (G1, G2) sampled at 115 d were significantly enriched in ^15^N compared to control plants in every case (Tables 3, 4; Figure 4). Also, the δ^15^N values of plant tissues were significantly higher for heavily fertilized (G2) versus more lightly fertilized (G1) plants (Tables 3, 4). The difference in mean δ^15^N values between the G1 and G2 plant organs was fairly consistent: 6.2‰ for stalks and roots, 6.4‰ for leaves (at 115 d), 7.6‰ for grain, and 7.8‰ for anthers.

**Table 3:** Isotopic and elemental compositions of plant samples (mean±1σ).

Treatment	Tissue	Sampling Date	δ^15^N (‰, AIR)	δ^13^C (‰, VPDB)	%N	%C
C0	Leaf	30 d	−0.3±3.6	–	5.1±0.7	–
	Leaf	75 d	5.5±1.4	–	2.7±0.6	–
	Leaf	115 d	3.9±1.0	−15.7±0.8	1.1±0.3	39.5±2.3
	Grain	115 d	2.7±0.7	−12.9±0.1	2.4±0.3	42.5±1.4
	Anther	75 d	5.7±0.4	−14.0±0.4	2.4±0.4	47.0±2.3
	Root	115 d	3.6±0.9	−15.0±0.5	0.8±0.2	44.7±1.1
	Stalk	115 d	3.2±1.4	−15.2±0.2	1.0±0.5	47.0±1.8
G1	Leaf	30 d	−5.0±10.0	–	4.9±0.9	–
	Leaf	75 d	32.4±2.2	–	4.1±0.3	–
	Leaf	115 d	26.8±2.0	−15.1±0.5	2.6±0.7	40.6±4.7
	Grain	115 d	25.5±1.6	−14.1±0.8	2.5±0.2	45.8±2.8
	Anther	75 d	34.2±3.4	−13.5±0.4	3.2±0.1	48.4±2.1
	Root	115 d	33.1±4.1	−15.2±0.3	1.4±0.7	44.2±4.2
	Stalk	115 d	38.4±1.9	−15.4±0.5	2.9±0.8	36.4±2.6
G2	Leaf	30 d	6.0±4.3	–	5.7±0.4	–
	Leaf	75 d	38.2±0.9	–	4.8±0.2	–
	Leaf	115 d	33.3±2.7	−15.4±1.0	3.2±0.6	42.6±3.9
	Grain	115 d	33.1±2.8	−13.3±0.3	2.6±0.2	44.6±1.7
	Anther	75 d	41.8±2.6	−13.5±0.4	3.3±0.5	45.2±4.4
	Root	115 d	40.1±2.6	−14.5±0.6	2.1±0.7	41.4±1.8
	Stalk	115 d	44.7±0.8	−14.7±0.6	3.4±0.2	29.7±0.8

**Table 4:** Results of ANOVA for differences in isotopic and elemental tissue compositions between treatments.

Tissue	Treatment	G1				G2			
		δ^15^N (‰, AIR)	δ^13^C (‰, VPDB)	%N	%C	δ^15^N (‰, AIR)	δ^13^C (‰, VPDB)	%N	%C
Leaf 30 d	C0	0.707	–	0.889	–	0.096	–	0.347	–
	G1	**–**	–	–	–	0.171	–	0.176	–
Leaf 75 d	C0	**<0.001**	–	**0.008**	–	**<0.001**	–	**0.002**	–
	G1	–	–	–	–	**<0.001**	–	**0.002**	–
Leaf 115 d	C0	**<0.001**	0.509	**0.003**	0.884	**<0.001**	0.857	**<0.001**	0.414
	G1	–	–	–	–	**0.001**	0.819	0.357	0.686
Stalk	C0	**<0.001**	0.640	**<0.001**	**<0.001**	**<0.001**	0.249	**<0.001**	**<0.001**
	G1	–	–	–	–	**<0.001**	0.056	0.415	**<0.001**
Grain	C0	**<0.001**	0.066	0.760	0.092	**<0.001**	0.150	0.463	0.348
	G1	–	–	–	–	**<0.001**	0.221	0.869	0.632
Anther	C0	**<0.001**	0.118	**0.010**	0.746	**<0.001**	0.135	**0.006**	0.669
	G1	–	–	–	–	**0.017**	0.997	0.940	0.280
Root	C0	**<0.001**	0.746	0.227	0.958	**<0.001**	0.249	**<0.001**	0.163
	G1	–	–	–	–	**<0.001**	0.076	0.415	0.250

Values in boldface are statistically significant (*p*<0.05).

A growing body of literature has emerged in recent years demonstrating that organic fertilizers, specifically those derived from animal waste, can cause large ^15^N enrichments of plant tissues (Table 5). The δ^15^N values reported here for plants grown in guano-fertilized soils are significantly higher than any published δ^15^N values for plants grown on other organic fertilizers to date (Table 5), but comparable to δ^15^N values for plants growing in ornithogenic soils (Table 2). The higher δ^15^N values in the G1 and G2 compared to the C0 plants is the result of the uptake of ^15^N-enriched guano-derived nitrogen. Moreover, the significantly higher tissue δ^15^N values in the G2 compared to G1 plants reflects, at least in part, the greater availability of guano-derived nitrogen throughout the course of the experiment. This does not imply that guano-derived N was absent in the G1 treatment towards the end of the experiment, but it is possible that N immobilization had overtaken N mineralization, reducing the amount of guano-derived N available to the plants.

**Table 5:** Summary of studies examining the influence of organic fertilization on plant δ^15^N values.

Fertilizer	Fertilizer δ^15^N (‰, AIR)	Plant	Plant δ^15^N (‰, AIR)	Δ^15^N_fertilized–control_	Reference
Pig manure	13.9	Maize	7.7	+1.1	[102]
Various composts	17.4±1.2	Maize	17.7	+13.5	[196]
Various composts	17.4±1.2	Nightshade	13.4	+10.7	[196]
Various composts	17.4±1.2	Pepper	14.5	+9.8	[196]
Various composts	17.4±1.2	Mustard	16.3	+12.7	[196]
Various composts	17.4±1.2	Melon	13.3	+10.1	[196]
Various composts	17.4±1.2	Lettuce	13.5	+9.4	[196]
Various composts	17.4±1.2	Spinach	9.5	+3.9	[196]
Various composts	17.4±1.2	Beefsteak plant	19.9	+15.4	[196]
Various composts	17.4±1.2	Sesame	17.8	+12.1	[196]
Pig manure	16.9	Chrysanthemum	10.3	+3.5	[194]
Pig manure	16.9	Cabbage	13.3	+5.6	[194]
Sheep manure	–	Sweet pepper	10.0	–	[198]
Chicken manure	–	Sweet pepper	10.2	–	[198]
Horse manure	–	Sweet pepper	9.8	–	[198]
Livestock manure	8.7±0.2	Orange (pulp)	9.0	–	[132]
Poultry manure	8.6±0.3	Orange (pulp)	8.5	–	[132]
Livestock manure	8.7±0.2	Orange (juice)	8.5	–	[132]
Poultry manure	8.6±0.3	Orange (juice)	7.9	–	[132]
Pig manure	16.4	Chinese cabbage	12.5	+11.0	[195]
Mixed (Cattle+poultry manure)	16.7	Tomato	13.5	+10.2	[199]
Mixed (Cattle+poultry manure)	9.9	Tomato	7.9	+4.6	[199]

### Elemental Concentration in Plant Parts

There were significant differences in N content between fertilized and control plants, with fertilized plants tending to have significantly higher N (Tables 3, 4). There were no significant differences in C content between control and fertilized plants for all organs, with the exception of the stalks, which had significantly lower %C in the fertilized plants compared to the control, and in G2 compared to G1 plants.

In general, the differences in C and N content between fertilized and unfertilized plants can be attributed to the accumulation of proteins, particularly those related to the GS-GOGAT pathway, that assimilate NH_4_^+^ and amino acids. Free amino acids tend to accumulate unabated in plant tissues with increasing supply of N irrespective of source, although different amino acids may accumulate at different rates depending on plant species and N source [45], [77]–[80]. Moreover, many studies have noted an increase in proteins, such as GS, in plant tissue in accordance with increasing NH_4_^+^ supply [80], [81]. Thus, the relatively high N content of the organs of fertilized plants likely reflects the accumulation of these N compounds.

The two amino acids that dominate the free amino acid pool when plants are supplied with excess N are glutamine and arginine [77], [82]. Arginine, which has a very low C∶N ratio (6∶4), has been implicated as an important product for the accumulation of excess N, possibly as a buffering mechanism against NH_4_^+^ toxicity [45], [83], [84]. Again, the accumulation of high levels of arginine in NH_4_^+^-fed plants fits with the pattern observed in the G1, and particularly the G2 plants. The very high levels of N and low levels of C in the stalks of the fertilized plants (compared to the control) suggests that the stalk was the most important accumulator for metabolites produced from excess N.

A notable exception to the pattern of increased N with fertilization is the grain, for which there was no significant difference in N content between treatments (Table 4). Our results suggest that at different levels of N supply and plant N content, there was no preferential allocation of accumulated N to the grain, and N that was absorbed post-silking was probably not allocated to the grain. A similar pattern was observed by Ma and Dwyer [85], although it is important to bear in mind the variability among maize hybrids in N metabolism during grain filling [86].

**Figure 5:**
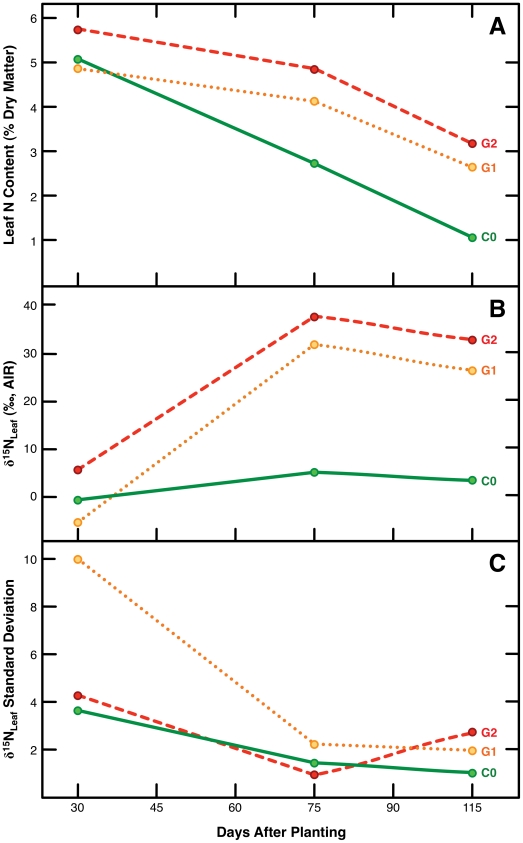
Temporal patterns in isotopic and element composition. (**A**) Leaf N content, (**B**) Leaf δ^15^N, and (**C**) standard deviation for Leaf δ^15^N.

As plants progress through various stages of growth, their uptake, metabolism and partitioning of N may change dramatically. In maize, a significant portion (45–65%) of the grain N is obtained from endogenous N reallocated primarily from the stalk and leaves, while the remaining grain N is obtained from uptake of exogenous soil N [87]–[90]. Leaf N content at 75 d and 115 d varied as a function of the amount of guano applied (ie. C<G1≤G2), although this was not the case for leaves sampled at 30 d, where there was no clear relationship between quantity of fertilizer applied and leaf N content (Figure 5a). This likely reflects both a reliance on stored seed N early in growth, and the short period of growth prior to transplanting (7 d) during which no fertilizer N was available.

We observed decreases in leaf N content over time, with leaf N content decreasing by 77.9% (C0), 46.9% (G1) and 47.1% (G2) between 30 and 115 d (Figure 5a). The maintenance of very high levels of N in G1 and G2 relative to C0 plants suggests the accumulation of plant N as a result of excess source N [91].

Based on the results of this study, seabird guano fertilization has the potential to significantly alter the C and N economy of maize plants. Specifically, fertilization results in increased N and decreased C∶N ratio in plant tissues, which likely arises because of increased accumulation of N–rich metabolites such as arginine, glutamine, and proteins related to NH_4_^+^ metabolism.

### Intraplant Variation in δ^15^N

Intraplant variability in nitrogen isotopic composition for all treatments was large, with maximum differences between mean organ δ^15^N being 3.0‰ for C0, 12.9‰ for G1 and 11.4‰ for G2 (Figure 4). We found significant differences in the δ^15^N values between maize plant organs for both control (*F*_4,20_ = 7.41, *p*<0.001) and fertilized (*F*_4,20_ = 18.60, *p*<0.001 for G1; *F*_4,20_ = 28.73, *p*<0.001 for G2) treatments (Figure 4). In all treatments, the grain possessed the lowest δ^15^N value, while anthers had the highest δ^15^N values in the control treatment and the second-highest δ^15^N values in the fertilized treatments, following stalks (Figure 4).

Significant variability in δ^15^N within plants has been recorded in several studies [30], [92]–[98]. Evans [99] suggests that, in general, plants with NO_3_^−^ as the primary N source are characterized by significant intraplant variability, while this is not true for plants with NH_4_^+^ as their primary N source. This general pattern results largely from the fact that NH_4_^+^ is assimilated into organic N only in the root, while NO_3_^−^ assimilation occurs both in roots and shoots (Figure 3B) [57], [100], [101]. Therefore, organics derived from NH_4_^+^ are assimilated from the same N pool in the roots, while NO_3_^−^ that has been translocated to the shoot prior to assimilation has already undergone some fractionation (in the roots) and is thus enriched in ^15^N [30], [95], [99].

The δ^15^N values of the roots were intermediate compared to other above-ground tissues, which does not fit with the scenario described above for NO_3_^−^ fed plants in which shoot tissues have higher δ^15^N values than roots. In the C0 and G1 plants, the roots did not differ significantly from stalks, grains, or leaves in terms of δ^15^N (Table 6). In the G2 plants, root δ^15^N was significantly lower relative to the stalk, but significantly higher than the leaf or grain (Table 6). The lack of a consistent pattern of root vs. shoot δ^15^N observed in this study likely reflects complex N metabolism, with relative reliance on NH_4_^+^ and NO_3_^−^, as well as guano-derived N changing over time.

**Table 6:** Results of ANOVA for differences in nitrogen isotopic composition between plant parts.

Treatment	Tissue	Leaf	Anther	Root	Stalk
C0	Grain	0.319	**<0.001**	0.626	0.908
	Leaf	–	**0.041**	0.981	0.803
	Anther	–	–	**0.013**	**0.004**
	Root	–	–	–	0.980
G1	Grain	0.915	**0.017**	0.077	**<0.001**
	Leaf	–	**0.035**	0.152	**<0.001**
	Anther	–	–	0.999	0.309
	Root	–	–	–	0.252
G2	Grain	0.999	**<0.001**	**<0.001**	**<0.001**
	Leaf	–	**<0.001**	**<0.001**	**<0.001**
	Anther	–	–	0.709	0.259
	Root	–	–	–	**0.022**

Values in boldface are statistically significant (*p*<0.05).

The relatively low grain δ^15^N values observed in this study are indicative of the reallocation of stored N. Choi et al. [102] also observed that grain tended to be depleted of ^15^N compared to stalks and leaves. This can be attributed to a kinetic isotope effect associated with catabolism and remobilization of stored plant N, which discriminates against ^15^N [103]. The high δ^15^N values of stalks suggest that this organ is an important source of accumulated N that is remobilized during grain filling. This supports the findings of Ta [104], who found that maize stalks functioned as a significant temporary storage reservoir for N-compounds. It is surprising that the leaves at 115 d are not characterized by higher δ^15^N values in comparison to the grain, as they are thought to be a significant contributor to grain N [105], [106]; this is discussed in more detail below. The importance of stalk, compared to leaf, N during grain filling may be specific to this variety of maize. Further study of the nitrogen metabolism of different maize hybrids is needed to clarify this issue.

### Temporal Variation in Plant δ^15^N Values

There was significant variability in maize leaves over the course of the experiment (Figure 5B). Maize leaves sampled at 115 d had lower δ^15^N values than those sampled at 75 d for all treatments; these differences were statistically significant for the fertilized groups, but not for the control group (Table 4). For all treatments, leaf δ^15^N values were significantly lower at 30 d compared to 75 d (Table 4).

Several studies have attempted to document changes in plant δ^15^N values over time and/or arising from natural leaf senescence. Kolb and Evans [97] and Garten [107] found no significant differences in the δ^15^N values of living and abscised leaves, which suggested a lack of ^15^N discrimination with N remobilization. Conversely, several other studies have found older or senescent plant leaves to be characterized by higher δ^15^N values, which has been attributed to a kinetic isotopic fractionation associated with N catabolism and reallocation [108]–[110]. We observed no significant difference between leaf δ^15^N at 75 d and 115 d for the control group, suggesting that under normal circumstances, there is no significant fractionation associated with N remobilization from leaves for this variety of maize. That there was a concurrent decrease in N content and δ^15^N for leaves between 75 and 115 d in the fertilized plants is counterintuitive, as the reallocation of leaf N to the grain should result in a ^15^N-enriched leaf. As was previously suggested for the stalk, we suspect that a significant portion of the leaf N pool consisted of accumulated N in the form of free amino acids (especially arginine and glutamine) as a result of high N supply and, in particular, high source [NH_4_^+^]. The reason that older or senescent plant parts are characterized by higher δ^15^N values is because the metabolic processes involved (e.g. deamination, transamination) are associated with large kinetic fractionations that concentrate the remaining substrate in ^15^N [111]. Therefore, if the majority of the decrease in leaf N between 75 and 115 d is the result of the transfer of organic N products (amino acids) to another part in the plant (e.g. the stalk), which is not associated with any known ^15^N fractionation [112], this would help to explain why the leaves are not relatively enriched in ^15^N at 115 compared to 75 d.

Leaf δ^15^N values were more variable at 30 d than at either 75 or 115 d (Figure 5c). This is likely a result of variable reliance on stored and absorbed N sources. Kolb and Evans [97] found that young leaves (*Quercus* and *Encelia*) had an isotopic composition (δ^15^N) that reflected both stored and absorbed N, while mature leaf δ^15^N values reflected primarily absorbed N. Very low leaf δ^15^N values (−12.4, −12.4, −10.2‰) were observed at 30 d for three of the guano-fertilized maize plants. These compositions probably arise from physiological responses to high soil [NH_4_^+^]. At high extracellular [NH_4_^+^], influx of NH_4_^+^ occurs only via a low-affinity transport system, with high-affinity transport system proteins being down-regulated; this process occurs in concert with the active efflux of NH_4_^+^ from the roots [34]. Yoneyama et al. [33] suggest that when NH_4_^+^ assimilation is slow (because extracellular [NH_4_^+^] is high), NH_4_^+^-N isotopic fractionation is larger, with relatively more ^15^N-enriched NH_4_^+^ being effluxed from the cell. Ariz et al. [34] found plants that were most sensitive to NH_4_^+^ toxicity also had the lowest tissue δ^15^N values. The fact that not all plants in the present study were characterized by low leaf δ^15^N values is difficult to explain, but may be the result of heterogeneous distribution of the guano throughout the soil or genotypic variability in resilience to NH_4_^+^ toxicity.

Temporal patterns in plant δ^15^N values are complicated and are determined by a number of factors. We suspect that significant changes in the N source occurred over time as a result of soil nitrification, and there were also significant changes in [source N] over time. This complication, however, is a reality of working with animal fertilizers, rather than hydroponic solutions, and must be taken into account when interpreting data from field settings.

### Guano Fertilization and Plant Carbon Isotopic Composition

We observed no difference in plant δ^13^C values resulting from guano fertilization for any of the organs analyzed (Tables 3, 4). In earlier studies, variable plant N sources have been associated with small, but significant variations in δ^13^C values [113]. It is thought that this association arises because different N sources (and different N source concentrations) may alter plant water-use efficiency and thus change the carbon isotope composition of plant tissues [114].

Previous studies have found plant δ^13^C values to be distinct in organic vs. inorganic fertilization regiments, an outcome ascribed to higher rates of soil microbiological activity [115], [116]. Specifically, Georgi et al. [116] suggest that CO_2_ released during decomposition is depleted of ^13^C. Because control and fertilized plants were grown in the same growth chamber, there would be no differences in the δ^13^C of CO_2_ utilized by either group of plants, although this may not be true for an agricultural field fertilized with guano. In general, the influence on nitrogenous fertilizers (both organic and inorganic) on plant δ^13^C is unclear. Experimental results have been conflicting, with studies finding δ^13^C values to increase [117]–[122], decrease [123], or be unaffected [120], [124] in response to N fertilization. The relationship between N fertilizer application and plant δ^13^C is likely mediated by several factors and warrants further study. We likely did not detect any difference in plant δ^13^C values resulting from fertilization because the magnitude of difference would be quite small [113] and our sample size was also quite small (*n* = 5 per treatment).

### Implications for Food Chemistry

Seabird guano is becoming increasingly popular as an organic alternative among farmers in the United States and Europe [5]. Moreover, as the demand for organically grown produce soars worldwide [125], there is an increased incentive for farmers in areas in close proximity to guano deposits (e.g. Peru, Ecuador, Chile, and Namibia) to use this fertilizer and market their produce as organic [5]. In recent years, there has been a surge in isotopic research directed at demonstrating isotopic distinctions between conventional and organically grown produce [126]–[136]. The reason that this technique may sometimes be effective is primarily that inorganic fertilizers tend to have δ^15^N values close to 0‰, while organic fertilizers tend to have higher δ^15^N values, although there is great variability (Table 2). Based on the results of this study, the application of seabird guano in an organic fertilization regime would result in a very large ^15^N enrichment of all plant tissues in comparison to unfertilized plants, or to plants treated with chemical fertilizers. The magnitude of this difference is much greater than what has been observed for other organic fertilizers (Table 5), and thus isotopic data would be useful in verifying use of seabird guano. Moreover, the very high δ^15^N value of the guano itself suggests that its presence in mixed organic fertilizers should also be detectable via isotope ratio mass spectrometry.

### Implications for Archaeology

Stable isotope analysis (δ^13^C and δ^15^N in particular) plays an increasingly important role in the reconstruction of prehistoric diet. Dietary reconstruction requires a thorough understanding of the sources of isotopic variation in the foods that were consumed [137]. Recently, the notion that animal manure may have influenced the δ^15^N values of plants grown in prehistoric Europe has been proposed [138]–[140] and integrated into regional paleodietary studies. In the Andean region, several fertilizers are thought to have been of some importance in prehispanic agriculture including llama dung [141] and seabird guano [9], [142], [143]. Based on the large settlements that developed on the coast of Peru (e.g. Moche, Chimú) and the relative infertility of local soils, Nordt et al. [8] have suggested that the application of some kind of nitrogenous fertilizer, possibly seabird guano, would have been necessary to maintain agricultural productivity in at least some parts of the region. Direct evidence for fertilization, however, is very difficult to come by. One of the primary goals of this study was to determine whether or not the enrichment in ^15^N resulting from guano fertilization would be sufficient to detect this agricultural practice in the isotopic composition of a human or animal consuming the fertilized plant. Based on the results of this study and others that have examined the biogeochemistry of seabird-associated sites (summarized in Table 2), the application of seabird guano to agricultural fields would have caused a significant increase in the δ^15^N value of plants and of animals consuming these plants. In archaeological bone collagen from western South America, high δ^15^N values are usually accompanied by high δ^13^C values. This pattern applies to both humans [144]–[146] and domestic animals [147], and has generally been attributed to the consumption of high trophic-level marine resources (e.g. predatory fish, marine mammals). Conversely, this pattern may also be caused by the consumption of maize (a C_4_ plant) fertilized with seabird guano, which appears (isotopically) very much like a high-trophic level marine organism. As such, it is important to be mindful of the possibility of guano-fertilization when interpreting diet, not just on the coast, but in the interior highland region as well. According to ethnohistoric documents, guano was moved great distances and prized by groups living in the highlands as an essential component in maize agriculture [9].

The Andes were certainly not the only region in which seabird guano was used extensively as a fertilizer. Millions of tonnes of guano were exported to Europe and North America during the nineteenth century and Peruvian seabird guano was the most highly prized fertilizer at that time [148]–[150]. Isotopic analysis is being employed with increased frequency within the context of historical archaeology [151]–[159], a period during which the possible influence of seabird guano must also be considered.

## Supporting Information

Table S1 **Raw isotopic and elemental data for all samples analyzed.**
(XLS)
