# Correction: Stable Isotope Biogeochemistry of Seabird Guano Fertilization: Results from Growth Chamber Studies with Maize (*Zea Mays*)

**DOI:** 10.1371/annotation/8e51b001-d65f-4331-a5df-9aa2c7d5454b

**Published:** 2012-08-16

**Authors:** Paul Szpak, Fred J. Longstaffe, Jean-François Millaire, Christine D. White

There was a typographical error in the title. The correct title is: Stable Isotope Biogeochemistry of Seabird Guano Fertilization: Results from Growth Chamber Studies with Maize (*Zea mays*). The correct citation is: Szpak P, Longstaffe FJ, Millaire J-F, White CD (2012) Stable Isotope Biogeochemistry of Seabird Guano Fertilization: Results from Growth Chamber Studies with Maize (*Zea mays*). PLoS ONE 7(3): e33741. 

